# Effect of Combination Montelukast and Nasal Mometasone on Childhood Adenoid Hypertrophy

**DOI:** 10.22038/IJORL.2024.73906.3490

**Published:** 2024-03

**Authors:** Mohsen Jafari, Bahar Pourroshani, Kambiz Eftekhari, Armen Malekiantaghi, Parisa Ashournia, Alireza Shafiei

**Affiliations:** 1 Division of Infectious Diseases, Department of Pediatrics, Bahrami Hospital, Tehran University of Medical Sciences, Tehran, Iran.; 2 Department of Pediatrics, Bahrami Hospital, Tehran University of Medical Sciences, Tehran, Iran.; 3 Pediatric Gastroenterology and Hepatology Research Center, Division of Gastroenterology, Department of Pediatric, Bahrami Children Hospital, Tehran University of Medical Sciences, Tehran, Iran.; 4 Division of Gastroenterology, Department of Pediatrics, Bahrami Children Hospital, Tehran University of Medical Sciences, Tehran, Iran.; 5 Division of Allergy and Clinical Immunology, Department of Pediatrics, Bahrami Hospital, Tehran University of Medical Sciences, Tehran, Iran.

**Keywords:** Adenoid, Children, Mometasone Furoate, Montelukast

## Abstract

**Introduction:**

Adenoid hypertrophy is a common childhood disease; its standard treatment is adenoidectomy. The desire for medical management is increasing due to fewer complications and more convenience. The present study investigated the effect of adding oral montelukast to mometasone nasal spray in treating adenoid hypertrophy.

**Materials and Methods:**

This was a randomized, double-blind, placebo-controlled study conducted at a referral teaching hospital (Tehran, Iran) from September 2020 to September 2021. Children aged 2 to 14 years with clinical and radiological findings of adenoid hypertrophy were enrolled. Patients were randomly divided into two groups: mometasone nasal spray with oral montelukast (case group) or mometasone with placebo (control group). Then, the clinical scores were compared before and two months after the intervention.

**Results:**

Ninety-six patients completed the study [62.5% male (n=60)]. Of these, 51 were in the case and 45 in the control group. The clinical score in each group decreased significantly after the intervention (P<0.001), but the decrease in clinical score in the case group was not significantly different from the control (p=0.576).

**Conclusion:**

The results showed that the combination therapy with mometasone and montelukast has the same efficacy as mometasone and placebo in treating adenoid hypertrophy. Adding montelukast to mometasone has no additional effect.

## Introduction

The adenoid is a part of Waldeyer's lymphatic ring located in the posterior upper part of the nasopharynx (1). The adenoid is one of the first sites exposed to inhaled microorganisms and allergens and is one of the body's first lines of defense against them (2,3). Physiologically, the adenoid begins to grow from early childhood and reaches its maximum size around 7-10 years of age, and after that, its size gradually decreases (4). Adenoid hypertrophy (AHT) is one of the main causes of upper respiratory tract obstruction in children (5). The prevalence of AHT has been reported to vary from 3% to 70% in children and adolescents (5,6). It may cause several complications, including recurrent rhinosinusitis, otitis media, obstructive sleep apnea, facial growth abnormalities, speech problems, and impaired quality of life (3). The exact pathogenesis of AHT is unknown and appears to be multifactorial, including upper respiratory infections, gastroesophageal reflux, allergies, and genetic factors (7,8).

 Different diagnostic methods are used for adenoid hypertrophy, such as lateral neck radiography and nasal endoscopy (9).

Adenoidectomy is the standard treatment for adenoid hypertrophy but may be associated with general anesthesia and surgical complications. These complications can be prevented by choosing non-surgical alternative methods. For this reason, the trend towards medical treatments has increased (10).

Due to the significant anti-inflammatory effects of intranasal corticosteroids, they are one of the main options for managing adenoid hypertrophy instead of surgery (11).

Leukotrienes are inflammatory mediators involved in inflammatory reactions by acting on the cysteinyl leukotriene receptor-1 (12).

Montelukast is one of the leukotriene receptor antagonists used to treat some allergic diseases (12). Various studies have been conducted on the effectiveness of montelukast alone or in combination with nasal corticosteroids in treating adenoid hypertrophy, with conflicting results (10,12,13). Regarding controversies in the literature, we decided to evaluate the effect of oral montelukast added to nasal mometasone compared to nasal mometasone alone in treating children with adenoid hypertrophy. The superiority of our study compared to similar studies (6,10,12,13) was the use of a placebo in the control group, which made our study design double-blind and helped to reduce bias in the study results.

## Materials and Methods

*Study design*

In a randomized, double-blind, placebo-controlled trial, one hundred children aged 2 to 14 with clinical and radiological findings of adenoid hypertrophy were enrolled. This study was conducted in the allergy clinic of a referral teaching hospital from the Tehran University of Medical Sciences (Tehran, Iran) from September 2020 to September 2021. 

Patients with any craniofacial, neuromuscular, or syndromic disorders, acute respiratory infection in the last two weeks, antibiotic use in the last two weeks, a history of sensitivity to corticosteroids or antileukotrienes, and children who suffered from drug side effects or underwent adenoidectomy were excluded. 

To calculate the sample size regarding similar studies (6,10,12), (taking into account the power of the study (β) equal to 0.2 and the error of the first type (α) equal to 0.05), the minimum sample size for each group of 50 people was determined. Sampling was simple, and the Permuted-Block Randomization method was used for randomization. This way, the supervisor assigned each treatment group an A and B code. Then, using the software for creating random numbers, Google Random Generator, the obtained random numbers were assigned to each treatment block (even numbers to block AB and odd numbers to BA), and the treatment order was determined. In the beginning, the demographic information (including age, gender, height, weight, and body mass index), history of otitis media, sinusitis, chronic cough, eczema, food sensitivity, other allergies, contact with pets and family history of allergies and the score of adenoid hypertrophy symptoms were recorded in the checklist. The score of adenoid hypertrophy symptoms, including three items: snoring, shortness of breath, and nasal speech, was determined between 0 and 4 based on the statements of the patients or their parents [0 (no symptoms), 1 (rarely), 2 (occasionally), 3 (often) and 4 (almost always)]. Using lateral neck X-rays, a radiologist calculated and recorded the adenoid to nasopharynx ratio (A/N ratio). A/N ratios more than 2 SD higher than the mean value appropriate for the age group were considered enlarged adenoids (4,14). Clinical scores were evaluated and recorded in the checklist at the onset and end of the study. Patients were randomly divided into two groups. Both groups received nasal mometasone (50 μg/puff in each nostril) for two months. In addition to mometasone nasal spray, the children randomly received Montelukast 5 mg or placebo labeled A and B. The drug and placebo were in the same shape, size, color, and taste. Neither researchers nor patients were aware of the drug or placebo packaging. After completing the study, the contents of the packages were decoded. The follow-up lasted two months. The primary outcome was measuring the clinical symptoms score (including snoring, shortness of breath, and nasal speech).

*Ethical considerations*

Written informed consent was obtained before enrollment. The information remained confidential. This study followed the international principles governing clinical research (Declaration of Helsinki). The researcher afforded the cost of medicine and placebo, and no extra cost was imposed on the patients. This study was approved by the Research Ethics Committee of Tehran University of Medical Sciences (IR.TUMS. MEDICINE.REC.1397.196) and registered in the Iranian Clinical Trials Registry (ID: IRCT20180718040520N1).

*Statistical analysis*

Statistical analysis was done using SPSS software version 21.0 (SPSS, Chicago, IL). The normal distribution of data was examined by the Kolmogorov–Smirnov test. The Paired t-test and Independent t-test were used to compare within and between groups of normal data, respectively. Wilcoxon and Mann Witney U tests were used respectively for within and between-group comparison of data not normally distributed. The chi-squared test was used to compare qualitative variables. Analysis of covariance (ANCOVA) test was used to remove the effect of confounding variables. Predictor variables were measured using Linear and stepwise regression tests. 

The quantitative variables were reported as mean ± Standard Error of the Mean (SEM) and median and interquartile range, and qualitative variables as frequency (percent). A P-value ≤ 0.05 was accepted as a statistically significant difference. The intention-to-treat method was used to replace missing data.

## Results

**Fig 1:**
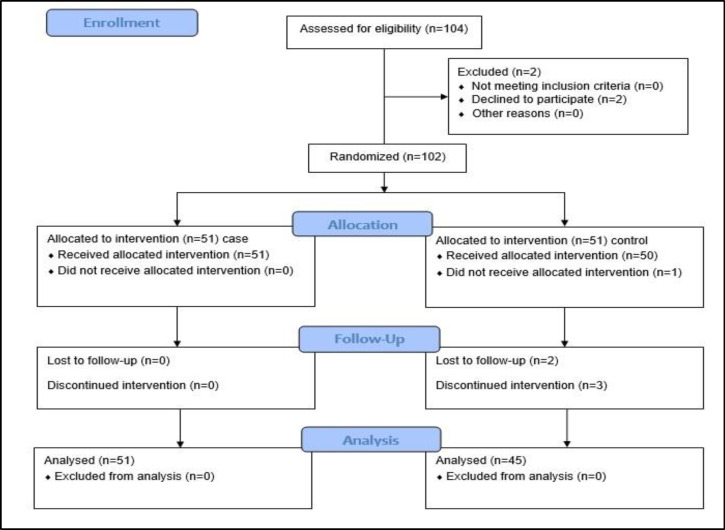
Study population (CONSORT Flow Diagram)

One hundred and four patients were enrolled, of whom 96 completed the study [62.5% male (n=60), age range 2-14 years]. Fifty-one children were included in both groups, and two refused to participate. Details of excluded cases are shown in Figure 1. Demographic and clinical characteristics of patients in both groups before the intervention are shown in Table 1. 

**Table1:** Demographic and clinical characteristics of patients in case (n=51) and control (n=45) groups before the intervention

**Variable**		**Control group (n=45)**	**Case group (n=51)**	**P-value**
Age*		7.4±2.8	6.4±2.3	0.083
Gender	Male	29 (64.4%)	31 (60.8%)	0.833
	Female	16 (35.6%)	20 (39.2%)	0.487
BMI*		17.4±3.9	18.0±4.5	0.637
History of the otitis media		10 (22.8%)	16 (31.4%)	0.363
History of chronic cough		15 (33.3%)	24 (47.1%)	0.213
History of sinusitis		9 (20.0%)	9 (17.6%)	0.799
History of eczema		5 (11.1%)	5 (9.8%)	1.00
History of cow's milk allergy		3 (6.7%)	7 (13.7%)	0.327
History of other allergies		7 (15.6%)	7 (13.7%)	1.00
History of receiving mometasone		10 (22.2%)	9 (17.6%)	0.616
Family History of Allergy		24 (53.3%)	20 (39.2%)	0.219
Nasal speech^¥^		3 (2-4)	3 (2-3)	0.453
Mouth breathing^¥^		4 (3-4)	3 (3-4)	0.536
Snoring^¥^		4 (2-4)	3 (3-4)	0.788
Clinical score*		9.1±2.1	8.9±1.9	0.576
A/N ratio*		0.80±0.10	0.80±0.12	0.896

*. Mean ± standard deviation ¥. Median (interquartile range) NS=Not significant

**Fig 2:**
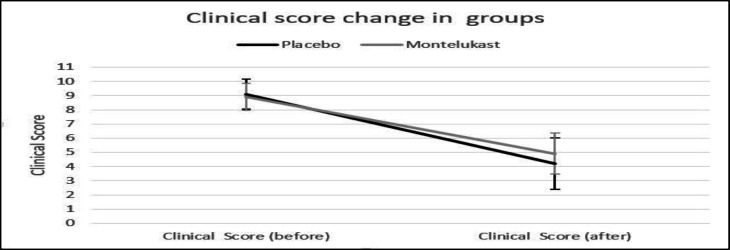
Changes in clinical score in the Montelukast and placebo groups

In terms of nasal speech, mouth breathing, and snoring, a significant reduction in the severity of symptoms was seen after the intervention in both case and control groups (The p-value in all three variables was less than 0.001), but in the comparison between the groups, this difference was not significant (p-value=0.376, p-value=0.306, p-value=0.246, respectively). The clinical score in both groups decreased significantly after the intervention (P-value<0.001). The decrease in clinical score in the case group was not significantly different from the control group (P=0.117) Figure 2. 

The final clinical score after adjustment of possible confounding variables such as age, gender, radiographic score, and clinical score before the intervention was compared using the ANCOVA test. There was no significant difference between the groups after adjustment (P=0.161). Statistical analysis using Linear Regression test showed that variables including age, A/N ratio, history of the otitis media, sinusitis, eczema, cow's milk protein allergy, other allergies, family history of allergy, and history of receiving mometasone did not have a significant predictive effect on the clinical score after treatment. Gender, chronic cough, and clinical score had a significant effect before the intervention. The clinical score before the intervention and male gender were associated with an increase in the clinical score after the intervention and a history of chronic cough with a decrease in the clinical score after the intervention (Table 2).

**Table 2:** Variables predicting clinical score after intervention

**Variable**	**Unstandardized Coefficients**		**Standardized Coefficients**		
	**B**	**Std. Error**	**Beta**	**t**	**P-value**
Constant	-.354	2.808		-.126	.900
Clinical score before the intervention	.591	.200	.357	2.950	.005
Chronic cough	-1.705	.784	-.265	-2.175	.034
Gender	1.682	.832	.247	2.022	.048

Adj.R^2^: 0.217

R^2^:0.261

R:0.511

## Discussion

Adenoid hypertrophy is a common disease in children. The common treatment is adenoidectomy. Due to surgery complications, the desire for medical management has increased. This study investigated the effect of adding oral montelukast to mometasone nasal spray in treating adenoid hypertrophy. Based on our results, mometasone improved the clinical symptoms, while adding montelukast to mometasone did not have an additional effect compared to placebo. Intranasal corticosteroids are one of the common treatments for adenoid hypertrophy due to their significant anti-inflammatory effects (11,15). 

In a meta-analysis by Chauhan et al. regarding the effects of intranasal mometasone in children with adenoid hypertrophy, 87 studies were evaluated (16). Their results showed that nasal mometasone effectively reduces adenoid size and other symptoms, similar to our results, which showed a reduction in symptoms after mometasone intake. Several studies have evaluated the effectiveness of Montelukast as an anti-inflammatory agent in treating adenoid hypertrophy (6,17,18). In the studies of Goldbart et al., Shokohi et al., and Khairandish et al., the improvement of AHT symptoms by Montelukast alone has been reported. Due to the anti-inflammatory effect of nasal corticosteroids and anti-leukotrienes, several studies have investigated the effect of drug combination therapy, compared to treatment with each one alone, in improving AHT symptoms (10,12,13,19-21). In most studies, in contrast to ours, adding montelukast to nasal corticosteroids improved efficacy compared to either drug alone (10,13,20,21). Tuhanıoğlu compared the efficacy of montelukast and mometasone in the treatment of AHT. Although both treatments were effective alone, the combination was not superior to monotherapy (12). The result of this study was similar to ours, meaning that adding montelukast to nasal mometasone did not have a superior effect. However, we did not investigate the effect of montelukast alone. 

In a non-blinded study by Alnori et al., the effect of mometasone and montelukast, alone or in combination, on AHT was compared (10). The results showed that monotherapy or drug combination effectively reduced symptoms after two months. Moreover, monotherapy with mometasone was more effective than montelukast. 

Contrary to our findings, the combination therapy showed a better result than monotherapy. A study conducted by Alnori showed no advantage of combination therapy over mometasone in reducing symptom recurrence one month after treatment discontinuation (10). We did not follow up on the patients after stopping the therapy, and it is expected that even if the follow-up had been done, the drug combination would not have had more advantages than monotherapy with mometasone. Khairandish depicted that the effect of treatment with nasal corticosteroids and montelukast on obstructive sleep apnea was higher in younger and non-obese children (19). In our study, variables such as a higher clinical score before the intervention and male gender were associated with a lower decrease, and the presence of chronic cough was associated with a higher decrease in the clinical score after the intervention.

## Conclusion

Our results showed that adding montelukast to mometasone nasal spray in children with adenoid hypertrophy has no advantage over mometasone alone.

### Limitations and suggestions

Although our study had strengths, such as having a control group, using a placebo, and being double-blind, it also had some limitations. It should be emphasized that our study was single-centered with a relatively small sample size. These findings need to be confirmed in studies with a larger sample size. Also, the effect of primary variables, such as allergic history, age of patients, presence of viral infection during treatment, and other factors on the effectiveness of different treatments needs to be further investigated.
